# Plasma Metabolomics Profiling of Metabolic Pathways Affected by Major Depressive Disorder

**DOI:** 10.3389/fpsyt.2021.644555

**Published:** 2021-09-27

**Authors:** Yue Du, Jinxue Wei, Zijian Zhang, Xiao Yang, Min Wang, Yu Wang, Xiongwei Qi, Liansheng Zhao, Yang Tian, Wanjun Guo, Qiang Wang, Wei Deng, Minli Li, Dongtao Lin, Tao Li, Xiaohong Ma

**Affiliations:** ^1^Psychiatric Laboratory and Mental Health Center, West China Hospital of Sichuan University, Chengdu, China; ^2^West China Brain Research Center, West China Hospital of Sichuan University, Chengdu, China; ^3^College of Foreign Languages and Cultures, Sichuan University, Chengdu, China

**Keywords:** major depressive disorder, metabolomics, metabolic pathway, glycine and serine metabolism, anxiety

## Abstract

**Background:** Major depressive disorder (MDD) is a common disease which is complicated by metabolic disorder. Although MDD has been studied relatively intensively, its metabolism is yet to be elucidated.

**Methods:** To profile the global pathophysiological processes of MDD patients, we used metabolomics to identify differential metabolites and applied a new database Metabolite set enrichment analysis (MSEA) to discover dysfunctions of metabolic pathways of this disease. Hydrophilic metabolomics were applied to identify metabolites by profiling the plasma from 55 MDD patients and 100 sex-, gender-, BMI-matched healthy controls. The metabolites were then analyzed in MSEA in an attempt to discover different metabolic pathways. To investigate dysregulated pathways, we further divided MDD patients into two cohorts: (1) MDD patients with anxiety symptoms and (2) MDD patients without anxiety symptoms.

**Results:** Metabolites which were hit in those pathways correlated with depressive and anxiety symptoms. Altogether, 17 metabolic pathways were enriched in MDD patients, and 23 metabolites were hit in those pathways. Three metabolic pathways were enriched in MDD patients without anxiety, including glycine and serine metabolism, arginine and proline metabolism, and phenylalanine and tyrosine metabolism. In addition, L-glutamic acid was positively correlated with the severity of depression and retardation if hit in MDD patients without anxiety symptoms.

**Conclusions:** Different kinds of metabolic pathophysiological processes were found in MDD patients. Disorder of glycine and serine metabolism was observed in both MDD patients with anxiety and those without.

## Introduction

Major depressive disorder (MDD) has been the leading cause of disability globally, affecting over one tenth of the world's population (1, 2). Even today, however, it is still challenging for us to assess MDD patients using an objective and established diagnostic method (3) due to huge heterogeneity in both clinical details and etiopathology of this disease (4). Unfortunately, failure in early diagnosis and treatment of MDD can lead to a higher rate of suicide and relapse (5). Therefore, it is essential to investigate the potential biomarkers and pathophysiological progress of MDD.

Former researches identifying biomarkers of MDD have indicated that metabolic disturbances may be correlated with the pathophysiology of MDD (6). Promising results from a study on drug-naïve patients with MDD indicated that their glucose levels had already been much higher than those of healthy controls when first diagnosed, and researchers concluded that the dysfunction of glucometabolic may result from MDD through hypercortisolemia (7). Accumulating evidence suggested that hypothalamic-pituitary-adrenal (HPA)-axis may play a critical role in the onset of MDD, and the disorder of this axis may also contribute to metabolic syndromes in depressive individuals (8). Some previous study had indicated the disturbance of amino acid metabolism in MDD, including high levels of glutamate, lysine, aspartate, serine, and so on (9). Moreover, a number of metabolic disturbances have been reported. The dysfunction of tryptophan pathway, kynurenine pathway, and more pathways (10–12), except classic metabolic pathways of neurotransmitters (13), was proved to be associated with MDD. Nevertheless, until now, findings on metabolic dysfunction in MDD are not consistent for many reasons such as pleomorphism of this disease and defects in methodological sources (14–16). Therefore, the existing evidences are hardly applicable to investigating the function of metabolic disorder in the MDD pathogenesis.

MDD patients may also have symptoms of anxiety (17, 18), which predict a longer disease course, higher level of depression, greater risk of suicide, and more possibility of treatment nonresponse (19–21). Therefore, it is necessary to investigate the difference of pathophysiological progress between MDD patients with anxiety symptoms (MDD-A) and MDD patients without anxiety symptoms (MDD-T). Previous researches had indicated that MDD patients with anxiety symptoms were more liable to present metabolic dysfunction. Data from a large, community-based study showed that a group of patients who had severe depression and anxiety symptoms showed higher HbA_1c_ levels and a greater need for insulin than those who showed no anxiety symptoms (22). In addition, another research showed that, in MDD patients, the elevated thyroid peroxidase levels indicated a higher level of anxiety symptoms (23). Therefore, the metabolic difference between MDD patients with and without anxiety symptoms should be further studied.

Metabolomics, which has been defined as the quantitative detection method of the global metabolic response of multiple living systems to environmental influence or other changes in the body, has recently been widely used to investigate the potential pathophysiological mechanisms (24). A majority of researchers focused on the application of metabolomics to mental illnesses studies have identified potential diagnostic biomarkers for MDD (25). In a systematic review focusing on the development of metabolomics in affective disorders showed that over 249 metabolites were found dysregulated in MDD. Moreover, almost half of those metabolites were reported in more than two studies (26). However, the search for biomarkers has been hindered by many reasons such as methodological differences (14). Studies suggested diagnostic systems such as metabolic set may contribute to better diagnostic efficacy (13).

Enrichment analysis and multivariate method have been widely used in omics researches, and omics-related databases, such as KEGG and DAVID, are built to identify pathways. Nevertheless, these databases are mainly used in genetic research (16); enrichment analysis in metabolomics research needs more professional databases. Metabolite set enrichment analysis (MSEA) has enabled researchers to identify and interpret patterns of mammalian (mainly human) metabolite concentration alterations in a biological context (27). Quantitative enrichment analysis (QEA), which is one of analytic methods of MSEA, can help provide information on pathways with global compounds that are identified when even just one of the compounds is significantly changed or when multi compounds are only slightly but consistently and exactly altered. Hence, more metabolic information could be kept (28).

In the present research, we applied liquid chromatography-mass spectroscopy (LC-MS) to identify plasma metabolites in MDD and healthy controls (HCs), and used MSEA to analyze the quantities of metabolic data in MDD.

## Materials and Methods

### Participants

This study recruited 155 right-handed participants from the Mental Health Center of West China Hospital, Sichuan University, consisting of 55 MDD patients and 100 sex-, gender-, and BMI-matched healthy controls (HCs). Permission was granted by West China Hospital of Sichuan University. All procedures in this research were designed and carried out in accordance with the guidelines issued by the Ethical Committee of Sichuan University. And all assessments were carried out after the participants and their legal guardians signed the informed consent forms. All patients were diagnosed as having major depressive disorder (MDD) according to the Diagnostic and Statistical Manual of Mental Disorders, Fourth Edition (DSM-IV). Then, we assessed the severity of depression and of anxiety using the 17-item Hamilton Depression Rating Scale (HAMD-17) and the 14-item Hamilton Anxiety rating Scale (HAMA-14), respectively. The total scores of HAMD-17 in all MDD patients involved in the present study were above 17. Depressive symptoms were then evaluated in five dimensions of HAMD, namely, anxiety, weight, cognition, retardant, and sleep disorder. Two dimensions of HAMA were also evaluated, namely, somatic anxiety and psychological anxiety (29, 30). Participants were excluded if they had (1) endocrine diseases, metabolic disorders, or receiving hormone medication; (2) any serious physical diseases; (3) other psychiatric disorders, such as dementia, schizophrenia, and substance abuse; (4) obvious psychosocial factors; or (5) any psychotropic medications during the past 12 weeks.

### Plasma Sample Detection

We collected venous blood of recruited participants in anticoagulated EDTA tubes on their first day of taking part in our research. Venipuncture for blood was set at 10:00 am or 4:00 pm. Plasma samples were stored at −80°C within 1 h after collection.

Then, liquid nitrogen was used to ground plasma samples individually; the homogenate was resuspended by being well vertexed with pre-chilled methanol as well as 0.1% formic acid. During the following 5 min, the samples were incubated on ice to keep the temperature low, and were then centrifuged at 15,000 rpm, 4°C. After that, liquid chromatography-mass spectroscopy (LC-MS) grade water was applied to some of the supernatant which had been diluted to the final concentration that contained 60% methanol. Subsequently, the samples were transferred to a fresh Eppendorf tube with 0.22 μm filter and were centrifuged at 15,000 *g*, 4°C for 10 min. The LC-MS/MS system analysis was injected with the filtrate. Finally, LC-MS/MS analyses were performed using a Vanquish UHPLC system (Thermo Fisher) as well as an Orbitrap Q Exactive HF-X mass spectrometer (Thermo Fisher). By using the 16-min linear gradient at a flow rate of 0.2 ml/min, we injected the plasma of participants onto a Hyperil Gold column (100 × 2.1 mm, 1.9 μm). Both eluent A (0.1% FA in Water) and methanol B were the positive polarity mode of the eluents. The solvent gradient was set as follows: 2% B for 12.0 min; 100% B for 14.0 min; 100–2% B for 1.5 min; 2–100% B for 14.1 min; and 2% B for 16 min. Then, we operated Q Exactive HF-X mass spectrometer in bipolarity modes with the help of capillary temperature at the temperature of 320°C, spray voltage of 3.2 kV, aux gas flow rate of 10 arb., and sheath gas flow rate of 35 arb. We processed the raw data files generated by UHPLC-MS/MS using the Compound Discoverer 3.0 for to detect and quantify performance peak. We then set the main parameters, including actual mass tolerance, minimum intensity, retention time tolerance, signal/noise ratio, and signal intensity tolerance. Following this step, we normalized peak intensities to the total spectral intensity. To forecast the molecular formula based on additive ions, normalized data on fragment ions and molecular ion peaks were used. During metabolite detection, QC samples, which were equally mixed experimental samples, were used to balance LC-MS system and monitor the status of the instrument.

Finally, the raw data files generated by LC-MS system were processed using the Compound Discoverer 3.0 (CD 3.0, Thermo Fisher) to perform peak alignment, peak picking, and quantitation for each metabolite. The main parameters were set as follows: retention time tolerance, 0.2 min; actual mass tolerance, 5 ppm; signal intensity tolerance, 30%; signal/noise ratio, 3; and minimum intensity, 100,000. After that, peak intensities were normalized to the total spectral intensity. The normalized data were used to predict the molecular formula bases on additive ions, molecular ion peaks, and fragment ions. Then, peaks were matched with the mzCloud (https://www.mzcloud.org/) and ChemSpider (http://www.chemspider.com/) database to obtain the accurate qualitative and relative quantitative outcomes.

### Statistical Analysis

First, to check inter-batch bias during metabolite detection, QC samples were evaluated using principal component analysis (PCA). Then, to alleviate the influence of environment, metabolites were identified using the human metabolome database (HMDB), and those that could be found in endogenous human bodies were included. The endogenous metabolites were normalized for subsequent analyses using log transformation and auto scaling, and SIMCAP+ 14.0 software was used for further analyses. The samples were used to build PCA model, and then outliers in these samples were eliminated. After that, the remaining samples were used to build the partial least squares-discriminant analysis model (PLS-DA), which was applied to explore the metabolic differences between HCs and depressive patients (31). Meanwhile, the samples of patients with and without anxiety symptoms were used to explore metabolic differences between MDD patients and HCs. Finally, to identify the possible biological functions of these metabolites responsible for MDD, metabolite set enrichment analysis (MSEA) was used to analyze the metabolic pathways in the online software MetaboAnalyst 5.0. *P*-value was set at <0.05 for metabolites resulting from Bonferroni method (HOLM), and the number of hits ≥2 was deemed as greatly enriched and being able to identify biological processes that warranted further investigation (32). Metabolites found in pathways of MDD with or without anxiety symptoms and dimensions found in HAMD and HAMA were analyzed respectively using spearman's rank correlation (33).

## Results

### Clinical Information of Participants

This study included 55 MDD patients and 100 healthy controls (HCs), and no demographic difference was observed between them. The MDD group was further divided in to two subgroups based on their total scores of HAMA: MDD with anxiety symptoms (*n* = 35, HAMA scores equal to or higher than 14) and MDD without anxiety symptoms (*n* = 20). No difference in demography was observed between these two subgroups, either. Demographic and clinical characteristics of all participants were summarized (Table 1 and Supplementary Table 1).

**Table 1:** Demographic and clinical characteristics of participants.

**Variables**	**MDD**	**HCs**	***p*** **value**
Sample size	55	100	-
Sex (male/female)	14/41	39/61	0.11
Age (year)	25.53 ± 8.19	25.08 ± 9.00	0.62
BMI	20.74 ± 2.89	21.09 ± 2.73	0.49
HAMD-17 total scores	21.53 ± 5.48	-	-
HAMA total scores	15.29 ± 6.00	-	-

*HCs, healthy controls; MDD, major depressive disorder; HAMD, Hamilton Depression Rating Scale; HAMA, Hamilton Anxiety Rating Scale; BMI, body mass index*.

### Metabolite Overview

Altogether, 822 metabolites were found in the raw data using LC-MS detection. The PCA plot of QC samples showed that, during the whole test process, the LC-MS system was stable and convincing (Supplementary Figure 1). Then, HMDB database was used to identify the source of the metabolites, excluding those that could only be found in the natural environment. A total of 222 endogenous metabolites were identified for further analyses (Supplementary Table 2). After log transformation and auto scaling processing, Kolmogorov-Smirnov test was used to assess the normality of the processed data, over half of which did not follow a normal distribution. In the PCA model of all samples, five outliers were found, including three MDD samples and two HCs samples (Supplementary Figure 2).

### MDD Samples vs. Healthy Controls

**Figure 1:**
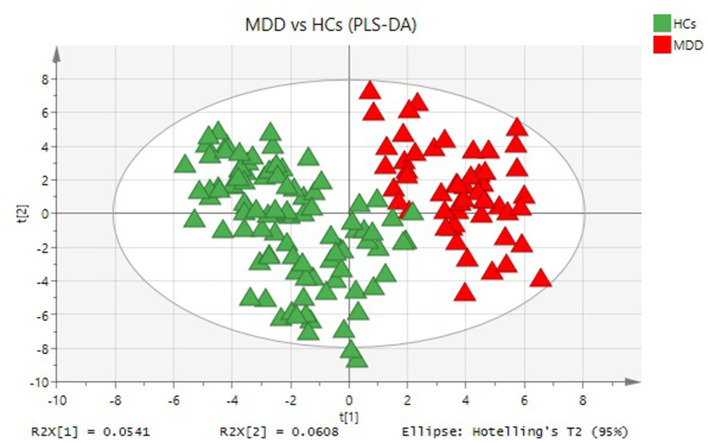
Metabolomic analysis of plasma samples from all participants. PLS-DA model indicates a significant difference between MDD (red triangle) and HCs (green triangle).

The PLS-DA plot score showed that MDD patients were clearly separated from HCs with little overlap (R^2^Y = 80%, Q^2^Y = 67%; Figure 1). To determine pathways in MDD patients different from those in HCs, MSEA analysis was performed, and then 17 pathways were significantly enriched in MDD patients (Table 2). In addition, 23 metabolites were hit in the pathways of MDD (Table 3).

**Table 2:** Differential metabolic pathways enriched from MDD group.

**Metabolite set**	**Total**	**Hits**	**Details**	**Holm P**
Ammonia recycling	32	3	L-glutamic acid; pyruvic acid; glutamine;	6.24E-05
Amino sugar metabolism	33	3	L-glutamic acid; pyruvic acid; glutamine;	6.24E-05
Glutamate metabolism	49	3	L-glutamic acid; pyruvic acid; glutamine;	6.24E-05
Urea cycle	29	4	L-glutamic acid; pyruvic acid; glutamine; ornithine	1.53E-04
Warburg effect	58	5	L-glutamic acid; pyruvic acid; glutamine; citric acid; D-erythrose 4-phosphate;	1.77E-04
Glycine and serine metabolism	59	6	L-glutamic acid; pyruvic acid; ornithine; 2-oxobutyric acid; betaine; creatine	1.86E-03
Fatty acid biosynthesis	35	4	decanoic acid; Lauric acid; cis-2-decenoic acid; beta-hydroxymyristic acid	2.16E-03
Purine metabolism	74	5	L-glutamic acid; glutamine; adenine; hypoxanthine; uric acid;	3.52E-03
Nicotinate and nicotinamide metabolism	37	2	L-glutamic acid; glutamine;	4.17E-03
Aspartate metabolism	35	2	L-glutamic acid; glutamine;	4.17E-03
Phenylacetate metabolism	9	2	L-glutamine; phenylacety lglutamine	4.37E-03
Cysteine metabolism	26	2	L-glutamic acid; pyruvic acid;	0.012
Alanine metabolism	17	2	L-glutamic acid; pyruvic acid;	0.012
Glucose-alanine cycle	13	2	L-glutamic acid; pyruvic acid;	0.012
Pyrimidine metabolism	59	3	dihydrothymine; glutamine; thymidine 5'-monophosphate;	0.015
Methionine metabolism	43	3	2-oxobutyric acid; betaine; 5'-S-Methyl-5-thioadenosine;	0.030
Androstenedione metabolism	24	3	androstenedione; testosterone; androsterone glucuronide	0.041

*Total: the total number of metabolites that can be hits in the pathway from MSEA; Hits: the total number of metabolites that hits in certain pathway; Details: the metabolites in our data that hits in the pathway; Holm P, p-value after Holm-Bonferroni adjusting*.

**Table 3:** Differential metabolites hit in differential pathways.

**Metabolites**	***P*** **-value**	**Fold change**	**VIP-value**
***Amino acids***			
L-glutamic acid	0.10	1.11	0.90
ornithine	0.77	0.96	0.39
phenylacetylglutamine	0.05	1.17	0.86
glutamine	9.71E-05	0.84	1.69
***Nucleobases***			
dihydrothymine	0.03	1.11	0.94
adenine	0.001	0.80	1.27
hypoxanthine	0.73	0.91	0.35
5'-S-Methyl-5'-thioadenosine	2.48E-04	0.79	1.23
thymidine 5'-monophosphate	0.73	0.92	0.25
***Other components***			
lauric acid	1.21E-036	1.06	1.24
citric acid	0.79	0.98	0.38
cis-2-Decenoic acid	5.44E-05	0.66	1.82
beta-Hydroxymyristic acid	0.002	1.16	1.10
pyruvic acid	0.002	0.84	1.56
uric acid	0.72	0.98	0.30
androstenedione	0.51	1.07	0.37
creatine	0.16	0.88	0.84
2-Oxobutyric acid	2.26E-04	1.19	1.36
betaine	0.04	0.93	0.37
D-Erythrose 4-phosphate	2.72E-04	0.68	1.32
decanoic acid	0.15	0.90	0.42
androsterone glucuronide	1.26E-04	1.49	1.63
testosterone	0.08	0.71	0.86

*P-values were derived from non-parametric Mann–Whitney U-test; Fold Change indicate higher or lower levels in patients; VIP-value (Variable Importance in the Projection) described the overall contribution of each metabolites to the PLS-DA model in component 1*.

### MDD Patients With and Without Anxiety Symptoms vs. HCs

The PLS-DA plot score also showed that metabolites we found could possibly distinguish anxious MDD patients, MDD patients lacking anxiety symptoms, and HCs (R^2^Y = 50%, Q^2^Y = 34%; Supplementary Figure 3). Then, using MESA analysis, 13 metabolic pathways were enriched in MDD patients with anxiety symptoms including ammonia recycling, amino sugar metabolism, glutamate metabolism, urea cycle, pyrimidine metabolism, Warburg effect, phenylacetate metabolism, nicotinate and nicotinamide metabolism, aspartate metabolism, fatty acid biosynthesis, purine metabolism, glycine and serine metabolism, and methionine metabolism (Supplementary Table 3A). Furthermore, we found 19 metabolites which were hit in those pathways of MDD (Supplementary Table 4A). In MDD patients without anxiety symptoms subgroup, only three metabolic pathways are significantly enriched including glycine and serine metabolism, arginine and proline metabolism, and phenylalanine and tyrosine metabolism (Supplementary Table 3B). Nine metabolites were hit in the pathways of MDD without anxiety symptoms (Supplementary Table 4B).

### Spearman's Rank Correlation

The metabolites found individually in two groups of MDD patients were analyzed using spearman' rank correlation associated with symptoms. In anxious MDD patient cohort, an inverse correlation was determined between adenine and weight in HAMD (r:−0.398, p = 0.022), and a great and inverse correlation was determined between hypoxanthine and psychological anxiety (r: −0.377, *p* = 0.031). 2-Oxobutyric acid (r: 0.362, *p* = 0.038) had also been found positively correlated with retardation in HAMD. In addition, in non-anxious MDD group, the highest positive correlations were determined respectively in L-glutamic acid (r: 0.718, *p* = 0.0005) and pyruvic acid (r: 0.679, *p* = 0.001) with retardation in HAMD. The L-glutamic acid (r: 0.491, *p* = 0.033) also had positive correlation with the level of depression.

## Discussion

In our study, the identified metabolic pathways were used to investigate the metabolic alterations in MDD individuals. Finally, 17 differential metabolic pathways were found in MDD group, and 23 metabolites were hit in those pathways. Dysfunction of glycine and serine metabolism was found in MDD patients in each subgroup, but dysfunction of arginine and proline metabolism and phenylalanine and tyrosine metabolism was only found in MDD patients without anxiety symptoms. In addition, pyruvic acid which was found in MDD patient group was related with the severity of depression. We noticed that L-glutamic acid, glutamine, and pyruvic acid had been hit in multiple pathways. Besides, L-glutamic acid in MDD patients lacking anxiety symptoms had correlation with retardation and the level of depression in HAMD.

In this research, we used quantitative enrichment analysis in MSEA to find metabolites and metabolic pathways that have biological functions. In previous studies, PCA, PLS-DA, and orthogonal least squares discriminant analysis (OPLS-DA) had been the most commonly used multivariate component methods in metabolomics analysis, but those approaches still had significant limitations (25). Those approaches usually focused on comparing the differences in metabolites production between different groups, focusing on a few significantly upregulated or downregulated metabolites, which tends to omit some metabolites with insignificant differentially produced but important biological significance. Nevertheless, QEA can help us find metabolic pathways with biological functions in diseases and metabolites with biological characteristics (34). In our study, the significantly biological changed L-glutamic acid, glutamine, pyruvic acid, and other metabolites were not found by the PLS-DA modal. However, quantitative enrichment analysis still viewed them as differential metabolites responsible for the discrimination between HCs and MDD patients, and metabolic pathways with biological functions were found. These results fully showed the advantage of QEA in finding more reproducible and more interpretable metabolites and pathways in untargeted metabolomics analysis (35).

Different kinds of metabolic pathophysiological processes were found in MDD patients. The 17 metabolite pathways that were identified in MDD group can be divided into four groups. Nine of the 17 pathways were associated with amino acid metabolism, 3 had correlation with nucleic acid metabolism, 3 had correlation with energy metabolism, 1 was related to lipid metabolism, and 1 was related to steroid metabolism. Previous studies showed that MDD had been a multifactorial disease, and environmental influence in MDD could not been ignored (36). For this reason, multidisciplinary theories, including neurotrophic alterations, neuroinflammation, neurovascular, and endocrine-immune system dysfunction, had attempted to explain the pathogenesis of MDD (37, 38). Our results are consistent with those previous studies. However, it is worth noticing that significantly disturbed amino acid metabolism was found in MDD individuals because most of the metabolic pathways are related to amino acid metabolism. Previous studies have mentioned that amino acids are important brain neurotransmitters (39), and they also showed that many amino acids may help the diagnosis of psychiatric diseases (39–41). In our study, we found the dysfunction of glycine and serine metabolism is consistent in MDD patients with or lacking anxiety symptoms.

Glycine or serine binding glutamate as co-agonist can help to activate N-methyl-D-aspartate receptors (NMDARs) (42), and the abnormality in NMDARs activity has been proven to result in both affective and cognitive disorder which is the core symptom of affective disorder by decreasing neuroplasticity (43). Reducing serine levels impairs NMDAR-mediated processes in hippocampus, prefrontal cortex, and amygdala and so on (44, 45), and these brain structures are closely related to mood disorders (46). Additionally, clinical studies demonstrated that ketamine, as a non-competitive NMDA receptor antagonist, has a rapid antidepressant response. Based on this, the new prodrug 4-Chlorokynurenine as a potent antagonist of NMDAR glycine agonist site was found to possess the potential of producing sustained and rapid antidepressant effect without the side effect profile of ketamine in MDD (47). Hence, NMDAR glycine site shows the potential to facilitate the development of antidepressant. However, the abnormality of NMDARs like NMDA/glycine-B site also contribute to anxiety symptoms. For instance, mutant mice that had reduction in NMDAR glycine affinity showed anxiety-like symptoms (48–50). Thus, we can conclude that the dyshomeostasis of glycine and serine metabolism can lead to both depressive and anxious symptoms.

The irregularity of arginine and proline metabolism was only found in the MDD group without anxiety symptoms. A double-blind, placebo-controlled crossover study on the mechanism of action of ketamine in depression clearly demonstrated that MDD patients had lower biological activity in arginine and proline metabolism, and that ketamine could modulate the bioavailability of arginine (51). Low level of arginine contributes to increased arginase activity which can lead to the severity of depression (52). Arginine-proline metabolism is connected with nitric oxide cycling (53). Arginine which is the precursor of proline is converted to nitric oxide and citrulline under the help of nitric oxide synthases (54). A recent research focusing on the influence factor of Alzheimer's disease indicated that NO is generated by NMDARS and is catalyzed neuronal nitric oxide synthase, and that high concentration of NO in brain is associated with the elevated IL-6 level and TNF-α which can also lead to cognitive disorder through the tau phosphorylation (55). Actually, our previous study on the relationship between the levels of inflammation cytokines and major depressive disorder had already found that MDD patients showed elevated IL-6 level (56). Therefore, arginine and proline metabolism may be involved in depressive symptoms by mediating inflammatory responses.

The disturbance of phenylalanine and tyrosine metabolism had been found in non-anxious MDD patients. Some previous study had indicated that MDD patients showed low levels of tyrosine, and after 4-week-treatment, tyrosine was significantly increased (57). Felger et al. (58) also found that compared with HCs, tyrosine metabolism was lower in MDD patients, and tyrosine metabolism was negatively associated with the level of C-reactive protein (CRP). They suggested that inflammation in patients could prevent the conversion of phenylalanine to tyrosine, and low level of tyrosine could induce the reduction of dopamine synthesis (59). A research focusing on the change of small RNA molecules in MDD indicated that the differentially expressed miRNAs in the brains of MDD patients could downregulate the expression of erb-b2 receptor tyrosine kinase 4, and influence phenylalanine and tyrosine metabolism (60).

Nevertheless, we did not find the disorder of arginine and proline metabolism, or phenylalanine and tyrosine metabolism in MDD patients with anxiety symptoms. We suspected that the anxiety symptoms may also impact arginine and proline metabolism in the opposite direction of MDD without anxiety symptoms.

We also noticed that L-glutamic acid, glutamine, and pyruvic acid had been hit in multiple pathways, indicating that these metabolites may be potential biomarker candidates for MDD. Higher level of glutamic acid was found in patients with mood disorders, compared with HCs and schizophrenia (61). Metabolic disorders in MDD patients could increase the burden of glutamate receptors in synapses, induce the release of glutamate in microglia cell, and reduce glutamate clearance, and thus interfering with glutamate transmission in the brain (62). Clinical studies have demonstrated that the abnormal L-glutamic acid and glutamine which are amino acid neurotransmitters in the brains of affective disorders' individuals contribute to changes in cortical excitability and inhibition (63, 64). Also, they had been discovered to influence energy metabolism as one of the tricarboxylic acids (TCA) cycle intermediates (65). Furthermore, in the study of relationship between metabolites and symptoms, we found a significantly high level of positive correlation between pyruvic acid and retardant/the level of depression in MDD. Pyruvate as the carboxylate anion of pyruvic acid is the end product of glycolysis and can further participate in TCA cycle which is the main process of energy metabolism (66). The low level of pyruvic acid had been found in MDD patients, and some studies suggested the interaction between amino acid metabolism and energy metabolism contributed to the onset of depression (67). However, even those metabolites may play an important role in MDD, we still lack evidence to validate a causal relationship between the discovered metabolites and MDD in this study. Further studies are needed to really raise a biomarker for MDD diagnosis.

Additionally, our study showed hypoxanthine was relatively associated with psychological anxiety, which suggested that the abnormality of purine metabolism and fatty acid metabolism may contribute to the severity of anxiety symptoms in MDD. A review of purinergic system changing in psychiatric diseases suggested that alterations in purine metabolism might be involved in anxiety disorder and depression because purinergic receptors are proved to be related to anxiety and depressive symptoms (68). A study on cell research implicated an important link between purine metabolic dysfunction in CD4+ T cells and anxiety-like behaviors (69). However, these results are mainly from rodent experiments. The instability of animal models and the differences between rodents and human beings may lead to these disagreements.

Despite the findings aforementioned, our study has several limitations. First, we did not include anxious patients lacking depressive symptoms; hence, the change of those pathways in this kind of patients was still unclear. Second, we did not control the fasting time of the enrolled participants. Although the interference of exogenous metabolites was excluded, our results still need further validation. Third, plasma was the only compartment used in this study to verify the identified pathways. Other kinds of samples, especially the cerebrospinal fluid, should be applied to validate our results. The phases of menstrual cycle in female participants were not taken into account in our study, but the influence of the cycle should also be noticed. Finally, we did not have a testing set to validate our results. Therefore, future researches should take those factors into consideration when identifying the potential pathways involved in major depressive disorder.

In conclusion, amino acid metabolism plays a key role in the pathophysiology of depression. The dysfunction of glycine and serine metabolism which is consistent in MDD patients with or lacking anxiety symptoms needs further investigation. Furthermore, the interaction between amino acid metabolism and energy metabolism may contribute to changes in the brains of MDD patients.

## Conclusions

In conclusion, different kinds of metabolic pathophysiological processes were found in MDD patients. The dysfunction of arginine and proline metabolism was observed only in MDD without anxiety symptoms, but the irregular of glycine and serine metabolism was found in both MDD with and without anxiety symptoms.

## Data Availability Statement

The original contributions presented in the study are included in the article/Supplementary Material, further inquiries can be directed to the corresponding author/s.

## Ethics Statement

The studies involving human participants were reviewed and approved by the Ethical Committee of Sichuan University. Written informed consent to participate in this study was provided by the participants' legal guardian/next of kin. Written informed consent was obtained from the individual(s), and minor(s)' legal guardian/next of kin, for the publication of any potentially identifiable images or data included in this article.

## Author Contributions

All authors have made significant scientific contributions to this manuscript. YD, XM, JW, and TL conceived and designed the experiments. YD, JW, ZZ, TL, XY, MW, YW, XQ, LZ, WG, QW, WD, ML, and XM performed the experiments. YD, JW, and ZZ analyzed the data. YD wrote the manuscript, and XM and DL reviewed the manuscript.

## Funding

We received funds from the National Natural Science Foundation of China (Grant No.: 81671344), Major International (Regional) Joint Research Project from National Natural Science Foundation of China (Grant No.: 81920108018), National Natural Science Foundation of China (Nos.: 82001432 and 81671344), China Postdoctoral Science Foundation (Nos.: 2020TQ0213 and 2020M683319), the 1.3.5 Project for Disciplines of Excellence, Special Foundation for Brain Research from Science and Technology Program of Guangdong (Grant No.: 2018B030334001), and West China Hospital of Sichuan University (Grant Nos.: ZY2016103 and ZY2016203) to support this study.

## Conflict of Interest

The authors declare that the research was conducted in the absence of any commercial or financial relationships that could be construed as a potential conflict of interest.

## Publisher's Note

All claims expressed in this article are solely those of the authors and do not necessarily represent those of their affiliated organizations, or those of the publisher, the editors and the reviewers. Any product that may be evaluated in this article, or claim that may be made by its manufacturer, is not guaranteed or endorsed by the publisher.
