# Supplementary material for: Plasma Metabolomics Profiling of Metabolic Pathways Affected by Major Depressive Disorder
Source: Front Psychiatry. 2021 Sep 27;12:644555. doi: 10.3389/fpsyt.2021.644555 (PMC8502978; doi:10.3389/fpsyt.2021.644555)
Supplement: Supplementary file 1 [file Data_Sheet_1.ZIP › supplementary material-revised/Table S4.docx]

**Table S4** Differential metabolites hit in differential pathways.

**A**

| metabolites | P-value | Fold Change | VIP-value |
| --- | --- | --- | --- |
| *Amino acids* |  |  |  |
| L-glutamic acid | 0.43 | 1.03 | 0.87 |
| ornithine | 0.99 | 0.98 | 0.39 |
| phenylacetylglutamine | 0.29 | 1.05 | 0.84 |
| glutamine | 1.38E-05 | 0.79 | 1.72 |
| *Nucleobases* |  |  |  |
| dihydrothymine | 0.01 | 1.15 | 0.96 |
| adenine | 0.002 | 0.79 | 1.28 |
| hypoxanthine | 0.93 | 0.92 | 0.35 |
| 5'-S-Methyl-5'-thioadenosine | 0.002 | 0.79 | 1.23 |
| thymidine 5'-monophosphate | 0.43 | 0.38 | 0.27 |
| *Other components* |  |  |  |
| citric acid | 0.82 | 0.99 | 0.37 |
| cis-2-decenoic acid | 2.81E-04 | 0.63 | 1.83 |
| beta-hydroxymyristic acid | 0.01 | 1.15 | 1.09 |
| pyruvic acid | 0.009 | 0.83 | 1.57 |
| uric acid | 0.46 | 1.03 | 0.27 |
| creatine | 0.78 | 1.00 | 0.79 |
| 2-Oxobutyric acid | 0.001 | 1.22 | 1.37 |
| betaine | 0.009 | 0.85 | 0.40 |
| D-erythrose 4-phosphate | 0.006 | 0.70 | 1.31 |
| decanoic acid | 0.25 | 0.93 | 0.42 |

**B**

| metabolites | P-value | Fold Change | VIP-value |
| --- | --- | --- | --- |
| *Amino acids* |  |  |  |
| L-glutamic acid | 0.04 | 1.25 | 0.87 |
| ornithine | 0.62 | 0.92 | 0.39 |
| tyrosine | 0.98 | 0.69 | 0.24 |
| D-proline | 0.27 | 1.19 | 1.21 |
| proline | 0.42 | 0.94 | 0.78 |
| *Other components* |  |  |  |
| pyruvic acid | 0.03 | 0.86 | 1.57 |
| creatine | 0.003 | 0.66 | 0.79 |
| 2-oxobutyric acid | 0.03 | 1.12 | 1.37 |
| betaine | 0.94 | 1.06 | 0.40 |

P-values were derived from non-parametric Mann–Whitney U-test; Fold Change indicated higher or lower levels in patients; VIP-value (Variable Importance in the Projection) described the overall contribution of each metabolites to the PLS-DA model in component 1. A. metabolites hit in the differential pathways of MDD with anxiety symptoms; B. metabolites hit in the differential pathways of MDD without anxiety symptoms.
