# Supplementary material for: Plasma Metabolomics Profiling of Metabolic Pathways Affected by Major Depressive Disorder
Source: Front Psychiatry. 2021 Sep 27;12:644555. doi: 10.3389/fpsyt.2021.644555 (PMC8502978; doi:10.3389/fpsyt.2021.644555)
Supplement: Supplementary file 1 [file Data_Sheet_1.ZIP › supplementary material-revised/Fig S2.docx]

**Fig S2.** Principal component analysis of all samples.


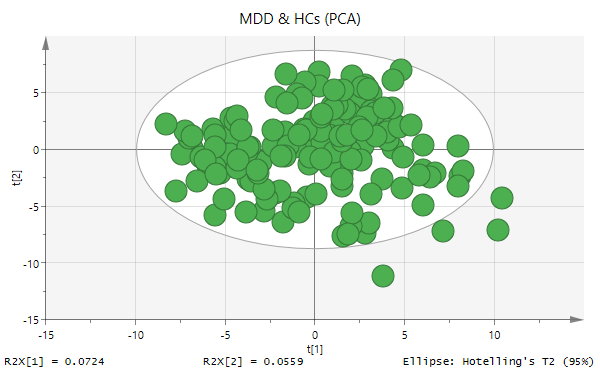


PCA analysis of all samples exhibits the overall status of metabolites.
