# Supplementary material for: Plasma Metabolomics Profiling of Metabolic Pathways Affected by Major Depressive Disorder
Source: Front Psychiatry. 2021 Sep 27;12:644555. doi: 10.3389/fpsyt.2021.644555 (PMC8502978; doi:10.3389/fpsyt.2021.644555)
Supplement: Supplementary file 1 [file Data_Sheet_1.ZIP › supplementary material-revised/Table S3.docx]

**Table S3** Differential metabolic pathways enriched from different groups.

**A**

| Metabolite Set | Total | Hits | Details | Holm P |
| --- | --- | --- | --- | --- |
| Ammonia Recycling | 32 | 3 | L-glutamic acid; pyruvic acid; glutamine; | 1.16E-04 |
| Amino Sugar Metabolism | 33 | 3 | L-glutamic acid; pyruvic acid; glutamine; | 1.16E-04 |
| Glutamate Metabolism | 49 | 3 | L-glutamic acid; pyruvic acid; glutamine; | 1.16E-04 |
| Urea Cycle | 29 | 4 | L-glutamic acid; pyruvic acid; glutamine; ornithine; | 2.95E-04 |
| Pyrimidine Metabolism | 59 | 3 | dihydrothymine; L-glutamine; thymidine 5’-monophosphate; | 9.75E-04 |
| Warburg Effect | 58 | 5 | L-glutamic acid; pyruvic acid; glutamine; citric acid; D-erythrose 4-phosphate; | 0.001 |
| Phenylacetate Metabolism | 9 | 2 | glutamine; phenylacetyl glutamine; | 0.003 |
| Nicotinate and Nicotinamide Metabolism | 37 | 2 | L-glutamic acid; glutamine; | 0.004 |
| Aspartate Metabolism | 35 | 2 | L-glutamic acid; glutamine; | 0.004 |
| Purine Metabolism | 74 | 5 | adenine; L-glutamic acid; hypoxanthine; uric acid; glutamine; | 0.008 |
| Fatty Acid Biosynthesis | 35 | 4 | lauric acid; decanoic acid; cis-2-decenoic acid; beta-hydroxy-myristic acid; | 0.02 |
| Glycine and Serine Metabolism | 59 | 6 | 2-oxobutyric acid; betaine; creatine; L-glutamic acid; ornithine; pyruvic acid; | 0.04 |
| Methionine Metabolism | 43 | 3 | 2-oxobutyric acid; betaine; 5'-S-Methyl-5-thioadenosine | 0.04 |

**B**

| Metabolite Set | Total | Hits | Details | Holm P |
| --- | --- | --- | --- | --- |
| Glycine and Serine Metabolism | 59 | 6 | 2-oxobutyric; betaine; creatine; L-glutamic acid; ornithine; pyruvic acid; | 0.004 |
| Arginine and Proline Metabolism | 53 | 5 | creatine; L-glutamic acid; L-proline; ornithine; D-proline; | 0.03 |
| Phenylalanine and Tyrosine Metabolism | 28 | 2 | L-glutamic acid; tyrosine | 0.05 |

Total the total number of metabolites that can be hits in the pathway from MSEA, Hits the total number of metabolites that hits in certain pathway, Details the metabolites in our data that hits in the pathway, Holm P p-value after Holm-Bonferroni adjusting. A. metabolic pathways enriched from MDD with anxiety symptoms with holm P below 0.05; B. metabolic pathways enriched from MDD without anxiety symptoms with holm P below 0.05.
