# Supplementary material for: Plasma Metabolomics Profiling of Metabolic Pathways Affected by Major Depressive Disorder
Source: Front Psychiatry. 2021 Sep 27;12:644555. doi: 10.3389/fpsyt.2021.644555 (PMC8502978; doi:10.3389/fpsyt.2021.644555)
Supplement: Supplementary file 1 [file Data_Sheet_1.ZIP › supplementary material-revised/Table S2.docx]

**Table S2** List of endogenous metabolites.

| metabolites | metabolites | metabolites | metabolites | metabolites |
| --- | --- | --- | --- | --- |
| 4-Nitrophenol | Docosahexaenoic acid | Deoxycholic acid | 3-Hydroxysebacic acid | 19-Nortestosterone |
| Methylimidazoleacetic acid | 2-Mercaptobenzothiazole | N-Acetyl-L-histidine | Azelaic acid | 3-Hydroxyvaleric acid |
| L-Glutamic acid | Gly-Leu | fMet | Hydrocinnamic acid | Kahweol |
| L-Tyrosine | Tripropionin | 4-Methoxycinnamaldehyde | DL-Tryptophan | Palmitoylcarnitine |
| Dihydrothymine | cis-2-Decenoic acid | N-Acetylcadaverine | Phenylacetylglutamine | 4-Hydroxybenzaldehyde |
| Lauric acid | 18-HETE | 5-Aminovaleric acid | D-Proline | 4-Hydroxyhippuric acid |
| Vanillin | Indole-3-lactic acid | 4-Ethylbenzaldehyde | Diphenol glucuronide | Androsterone glucuronide |
| 1-Naphthol | N2, N2-Dimethylguanosine | 4-Methoxycinnamic acid | Veratrole | 2-Furoic acid |
| Panaxytriol | Glycodeoxycholic acid | Glycocholic acid | Cortisone | 4-Pyridoxic acid |
| 2-Hydroxycinnamic acid | Paracetamol | 4-Trifluoromethylphenol | D-Malic acid | Pyrogallol |
| Tetradecanedioic acid | 4-Phenyl-3-buten-2-one | Undecanoic acid | Hexanoylcarnitine | 3-Acetyl-2,5-dimethylfuran |
| 3-Phenyllactic acid | Trifluoroacetic acid | Albendazole sulfoxide | Allantoic acid | p-Cresylsulfate |
| Glycoursodeoxycholic acid | Dihydropteroic acid | Indole-3-propionic acid | Homoarginine | 3-Indoleacetonitrile |
| Prostaglandin E1 | Docosapentaenoic acid | 1-Methyluric acid | Indole-3-acetic acid | Isophorone |
| L-Kynurenine | Theobromine | Alanyltyrosine | 4-Methylphenol | 1,7-Dimethyluric acid |
| Adenine | Sphingosine 1-phosphate | O-Desmethylvenlafaxine | Hippuric acid | 5-Hydroxytryptophan |
| Jasmonic acid | gamma-Glu-Gly | Normorphine | 1-Methylxanthine | Estrone sulfate |
| Ornithine | Cafestol | Caffeine | N-Cinnamoylglycine | Monobutyl phthalate |
| Indole-3-butyric acid | Formylkynurenine | 3-Methyladipic acid | beta-Hydroxymyristic acid | Testosterone |
| Citric acid | Tetrahydrocortisone | Vanillyl alcohol | Tiglic acid | Testosterone sulfate |
| 2-Methoxyestradiol | N-Acetyl-L-glutamine | Piperine | MG (16:1) | Cytosine |
| Paliperidone | Creatinine | Capryloylglycine | Celastrol | L-Pyroglutamic acid |
| Skatole | Cortisol | D-Maltose | 12-Hydroxydodecanoic acid | Phenobarbital |
| Cholic acid | Prostaglandin B1 | Haplopine | 3-Hydroxyisoheptanoic acid | Indolyl-3-acryloylglycine |
| Docosatrienoic acid | 5-Sulfosalicylic acid | Hexadecanedioic acid | Levothyroxine | Corchorifatty acid F |
| 4-Phenylbutyric acid | Retinoic acid | Betaine | gamma-Glu-gln | L-Norleucine |
| 1,5-Anhydro-D-glucitol | Diphenylamine | Tetrahydrocurcumin | Propionylcarnitine | 3-Methoxybenzaldehyde |
| Threonic acid | Pyruvic acid | Ketoleucine | Picolinic acid | N4-Acetylcytidine |
| Nicotinuric acid | N-Desmethylvenlafaxine | Oleamide | Cyclohexylamine | 2-Oxobutyric acid |
| Acrylic acid | Acetyl-L-carnitine | Tyrosine | Calcitriol | Coenzyme Q1 |
| N-Desmethyltramadol | Uric acid | P-Cresol glucuronide | 3-Hydroxydecanoic acid | D-Erythrose 4-phosphate |
| N-Acetyltryptophan | Androstenedione | Pipecolic acid | Desoxycortone | Pentadecanoic acid |
| Trigonelline | Monoolein | Benzylsuccinic acid | 10-Hydroxycarbazepine | Mevalonolactone |
| Decanoylcarnitine | Eicosapentaenoic acid | Arachidonic acid | Proline | 3-Indoxyl sulphate |
| Mycophenolic acid | Creatine | Asp-His | 1-Methylhistidine | Isobutyric acid |
| LPA (18:0) | cystine | cis,cis-Muconic acid | Palmitoleic acid | 4-Methoxybenzaldehyde |
| L-Saccharopine | N, N-Dimethylsphingosine | L-5-Hydroxytryptophan | Dihydrokawain | 2,6-Xylidine |
| Tridecylic acid | Theophylline | Propylparaben | Perindoprilat | LPA (18:0) |
| Corticosterone | Paraxanthine | Quebrachamine | Decanoic acid | Sulcatone |
| Avocadyne 1-acetate | Hypoxanthine | Cyclamic acid | Methyl indole-3-acetate | Glutamine |
|  |  |  |  |  |
| LysoPC(22:5(7Z,10Z,13Z,16Z,19Z)) | 2-Methyl-5-acetonyl-7-hydroxychromone | 3-Hydroxy-3-methylbutanoic acid | 4-Hydroxy-3-methoxymandelic acid | 1,5-Dihydroxy-3,4-dimethoxy-10-methyl-9(10H)-acridinone |
| 8Z,11Z,14Z-Eicosatrienoic acid | Eicosapentaenoic acid ethyl ester | Taurochenodeoxycholic acid | meta-O-Dealkylated flecainide lactam | 2-Isopropylmalic acid |
| 5'-S-Methyl-5'-thioadenosine | N6, N6, N6-Trimethyl-L-lysine | 16-Hydroxyhexadecanoic acid | Leukotriene B4 Ethanolamide | 2,6-Di-tert-butyl-1,4-benzoquinone |
| Thymidine 5'-monophosphate | 11H-Benzo[a]fluoren-11-ylacetic acid | 2'-Deoxyinosine 5'-monophosphate | Taurolithocholic acid 3-sulfate | Carbamazepine 10,11-epoxide |
| O-methoxycatechol-O-sulphate | 5-Hydroxyindole-3-acetic acid | Arachidonic acid methyl ester |  |  |
