# Supplementary material for: Plasma Metabolomics Profiling of Metabolic Pathways Affected by Major Depressive Disorder
Source: Front Psychiatry. 2021 Sep 27;12:644555. doi: 10.3389/fpsyt.2021.644555 (PMC8502978; doi:10.3389/fpsyt.2021.644555)
Supplement: Supplementary file 1 [file Data_Sheet_1.ZIP › supplementary material-revised/Table S1.docx]

**Table S1** Demographic and clinical characteristics of participants.

| Variables | MDD-A | MDD-T | HCs | p value | | | |
| --- | --- | --- | --- | --- | --- | --- | --- |
| Sample size | 35 | 20 | 100 | MDD-HCs | MDDA-MDDT | MDDA-HCs | MDDT-HCs |
| Sex（male/female） | 10/25 | 4/16 | 39/61 | 0.112 | 0.539 | 0.312 | 0.130 |
| Age(year) | 25.86±7.84 | 24.95±8.94 | 25.08±9.00 | 0.62 | 0.60 | 0.47 | 0.93 |
| BMI | 20.10±2.58 | 21.86±3.12 | 21.09±2.73 | 0.49 | 0.11 | 0.16 | 0.67 |
| HAMD | 22.17±5.49 | 20.40±5.43 | - | - | 0.25 | - | - |
| HAMA | 18.60±4.65 | 9.50±2.84 | - | - | 8.2E-10 | - | - |
| HCs healthy controls, MDD major depressive disorder, MDD-A major depressive disorder patients with anxiety disorder, MDD-T major depressive disorder patients without anxiety symptoms, HAMD Hamilton Depression Rating Scale, HAMA Hamilton anxiety Rating Scale, BMI body mass index | | | | | | | |
